# Global prevalence of polypharmacy and potentially inappropriate medication in older patients with dementia: a systematic review and meta-analysis

**DOI:** 10.3389/fphar.2023.1221069

**Published:** 2023-08-24

**Authors:** Mengnan Zhao, Zhaoyan Chen, Ting Xu, Ping Fan, Fangyuan Tian

**Affiliations:** Department of Pharmacy, West China Hospital, Sichuan University, Chengdu, Sichuan, China

**Keywords:** polypharmacy, potentially inappropriate medication, older, dementia, meta-analysis, factors

## Abstract

**Background:** Older patients with dementia always need multiple drugs due to comorbidities and cognitive impairment, further complicating drug treatment and increasing the risk of potentially inappropriate medication. The objective of our study is to estimate the global prevalence of polypharmacy and potentially inappropriate medication (PIM) and explore the factors of PIM for older patients with dementia.

**Methods:** We searched PubMed, Embase (Ovid), and Web of Science databases to identify eligible studies from inception to 16 June 2023. We conducted a meta-analysis for observational studies reporting the prevalence of potentially inappropriate medication and polypharmacy in older patients with dementia using a random-effect model. The factors associated with PIM were meta-analyzed.

**Results:** Overall, 62 eligible studies were included, of which 53 studies reported the prevalence of PIM and 28 studies reported the prevalence of polypharmacy. The pooled estimate of PIM and polypharmacy was 43% (95% CI 38–48) and 62% (95% CI 52–71), respectively. Sixteen studies referred to factors associated with PIM use, and 15 factors were further pooled. Polypharmacy (2.83, 95% CI 1.80–4.44), diabetes (1.31, 95% CI 1.04–1.65), heart failure (1.17, 95% CI 1.00–1.37), depression (1.45, 95% CI 1.14–1.88), history of cancer (1.20, 95% CI 1.09–1.32), hypertension (1.46, 95% CI 1.05–2.03), ischemic heart disease (1.55, 95% CI 0.77–3.12), any cardiovascular disease (1.11, 95% CI 1.06–1.17), vascular dementia (1.09, 95% CI 1.03–1.16), chronic obstructive pulmonary disease (1.39, 95% CI 1.13–1.72), and psychosis (1.91, 95% CI 1.04–3.53) are positively associated with PIM use.

**Conclusion:** PIM and polypharmacy were highly prevalent in older patients with dementia. Among different regions, the pooled estimate of PIM use and polypharmacy varied widely. Increasing PIM in older patients with dementia was closely associated with polypharmacy. For other comorbidities such as heart failure and diabetes, prescribing should be cautioned.

## 1 Introduction

The statistics of epidemiology revealed that in 2019, there were 703 million individuals aged 65 years or above living in the world, and the number was expected to reach 1.5 billion by 2050 (He and Kinsella, 2020). The global population aging would further accelerate the increase in the geriatric population, which imposed significant demands on the healthcare system. Meanwhile, increased medication use is one of the important challenges (Chiatti et al., 2012; Johnell, 2015).

Polypharmacy is defined as the concurrent use of multiple drugs, generally taking five or more drugs (Masnoon et al., 2017; Rankin et al., 2018). The older population often suffered multiple diseases, and polypharmacy was insufficient for controlling or curing diseases. A cross-sectional study performed by Chandrasekhar reported that the prevalence of polypharmacy in 210 inpatients aged 65 years or above was up to 60% and that of hyperpolypharmacy (ten or more drugs) was 35.7% (Chandrasekhar et al., 2019). Moreover, aging-related alteration in pharmacokinetics and pharmacodynamics might lead to stronger drug effects and prolongation of drug action time (Shi et al., 2008). Thus, the management of adverse effects on multiple drugs and potential drug–drug interaction among the older population was rather complicated and challenging.

Dementia, a degenerative nervous system disorder, features irreversible decline in cognitive function (Prince et al., 2013). Patients diagnosed with dementia were especially sensitive to adverse effects of central nervous system (CNS) drugs (Bell et al., 2012). Communication disorder caused by cognitive impairment and concomitant mental symptoms would lead to more complicated drug use in patients with dementia (Johnell, 2015). Furthermore, compared with non-dementia, dementia was more likely to be accompanied by other chronic diseases, such as hypertension and diabetes, exposing a higher risk of polypharmacy (Clague et al., 2017). Banta et al. indicated older patients with dementia are more likely to have five or more current prescriptions (Banta, 2017). Therefore, we should attach great importance to drug medications for older patients with dementia.

Potentially inappropriate medication (PIM) is an important concept to assess the quality of drug use. The term was first proposed by the American Panel in 1991, defined as those drugs with potential risks outweighing the benefits (Renom-Guiteras et al., 2015). A large number of studies have demonstrated PIM was associated with drug-related problems and adverse outcomes, such as increasing risk of hospitalization and death and incurring extra medical expenditure (Hagstrom et al., 2015; Hyttinen et al., 2017; Murphy et al., 2020). In order to evaluate PIM use and avoid the occurrence of adverse events, several explicit tools based on expert census were developed. A systematic review showed a total of 46 screening lists of PIM in the world that were identified, covering four continents and 13 countries (Kaufmann et al., 2014). The most frequently used criteria were Beers criteria and STOPP/START criteria (American Geriatrics Society Beers Criteria^®^ Update Expert Panel, 2019; O'Mahony et al., 2018), including drugs that should be avoided for treating common systemic diseases in elderly patients, possible adverse reactions, drug–drug interactions, drug–disease interactions, and risky drug use based on the renal function level. PIM use among older people was prevalent, especially in frail patients with dementia who need long-term care (Kristensen et al., 2018). Due to the difference of medical habits in each country and screening tools, the prevalence of PIM in patients with dementia varied widely. In European countries, 60% of older patients with dementia had at least one PIM based on the European Union (7)-PIM list (Renom-Guiteras et al., 2018). In China, the prevalence of PIM was 39.43% evaluated using 2019 Beers criteria (Zhao et al., 2022). Thus, a pooled analysis is necessary to conduct for evaluating the PIM and polypharmacy in patients with dementia, further providing a reference for countries that have not yet carried out a relevant study about drug burden in older patients with dementia.

To date, several reviews about PIM use and polypharmacy in dementia have been published (Johnell, 2015; Disalvo et al., 2016; Redston et al., 2018; Hukins et al., 2019; Delgado et al., 2020), but limited to a specific type of dementia or specific population (such as community or inpatients), or only qualitatively described the prevalence of PIM use or polypharmacy. In the systematic review and meta-analysis, we first summarized the pooled estimate of PIM and polypharmacy in older patients with dementia (not including mild cognitive impairment) across different regions and explored the association between PIM and polypharmacy and reviewed other factors associated with PIM use.

## 2 Methods

The study protocol of this systematic review and meta-analysis was registered on PROSPERO (CRD42022368310). The study was conducted based on MOOSE (Meta-analysis of Observational Studies in Epidemiology) guidelines (Stroup et al., 2000) and the Preferred Reporting Items for Systematic Reviews and Meta-Analyses guidelines (Moher et al., 2015).

### 2.1 Search strategy

A comprehensive search of PubMed, Embase (Ovid), and Web of Science was performed from inception to 16 June 2023. The search strategy was using a combination of Medical Subject Headings (MeSH) and free text words. The specific search details in different databases are listed in Supplementary Table S1. In addition, we performed manual searching of selected published full-text reviews, identifying other potential relevant articles.

### 2.2 Selection criteria

Studies were included if they recruited older adults with dementia (≥65 years, or mean age ≥70 years), reported the prevalence of polypharmacy (five or more) or PIM in dementia, used explicit criteria to identify PIM, and wrote the manuscript in English. The diagnosis of dementia was based on medical records, DSM criteria, ICD code, or other criteria. In addition, the study design of the included articles was observational studies (cross-sectional study or cohort study).

Studies were excluded if study subjects had mild cognitive impairment, or if those studies were conference abstracts, reviews, and comments.

### 2.3 Data extraction

Two reviewers (MN Zhao and ZY Chen) independently extracted and verified the data. We extracted information including study characteristics (first author, year of publication, and country), basic information of study subjects (age, sex, and sample size), and study design (setting, prevalence of polypharmacy or PIM, and explicit criteria to evaluate PIM). Any discrepancy between two reviewers was resolved by consultation with a third reviewer (FY, Tian).

### 2.4 Selection of studies

Two reviewers (MN Zhao and ZY Chen) independently screened the titles and abstracts of initially included literature according to the inclusion and exclusion criteria. The full text was further assessed if the eligibility of the study was not clearly determined from the abstract. Any inconsistency in the process of screening was resolved by consulting a third senior investigator (Ting Xu).

### 2.5 Quality assessment

The methodological quality of the cross-sectional study was evaluated using the Agency for Healthcare Research and Quality (AHRQ) (Rostom et al., 2004; Hu et al., 2015). A total of 11 items were listed in AHRQ, including 1) the source of data; 2) eligible criteria for study subjects; 3) time period for included population; 4) whether or not subjects were consecutive; 5) whether the outcome indicators are affected by other factors; 6) any assessments for quality assurance; 7) explanation for excluding any patients from the analysis; 8) measurements taken for controlling confounding factors; 9) description for the handing of missing data; 10) summary for patient response rate and completeness of data collection; 11) clarification of follow-up results. The highest score was 11, while the lowest score was 0. If the score was 8 or above, this study was considered high quality. If the score was 3 or below, this study was considered low quality. If the score was between 3 and 8, this study was considered medium quality. The methodological quality of the cohort study was evaluated by the Newcastle–Ottawa scale (Stang, 2010). If the NOS score ≥8, this study was considered high quality. If the NOS score ≤5, this study was considered low quality. If the NOS score was 6 or 7, this study was considered medium quality (Bedaso and Duko, 2022).

### 2.6 Statistical analysis

We applied STATA, version 16 (Stata Corporation, College Station, Texas, United States) to perform a meta-analysis for polypharmacy and PIM. The pooled prevalence estimate was reported as a proportion with 95% confidence intervals (CI). The I2 statistics was used to assess the magnitude of heterogeneity. When I2 >50%, heterogeneity among studies was considered large and the DerSimonian–Laird random-effect model was applied in analysis. In case of significant heterogeneity, subgroup analysis (e.g., regions, the proportion of females, criteria, and severity of dementia)) was performed to investigate the source of heterogeneity. We also estimated the 95% prediction interval, which further accounts for between-study heterogeneity and evaluates the uncertainty for the effect that would be expected in a new study addressing that same association (Higgins et al., 2009; Migliavaca et al., 2022). Furthermore, a pooled odds ratio was used to analyze the association between PIM and factors when two or more studies reported the same and adjusted odds ratio. Regarding the risk of publication bias, we adopted Egger’s and Begg’s tests for evaluation.

## 3 Results

### 3.1 Study selection

**FIGURE 1:**
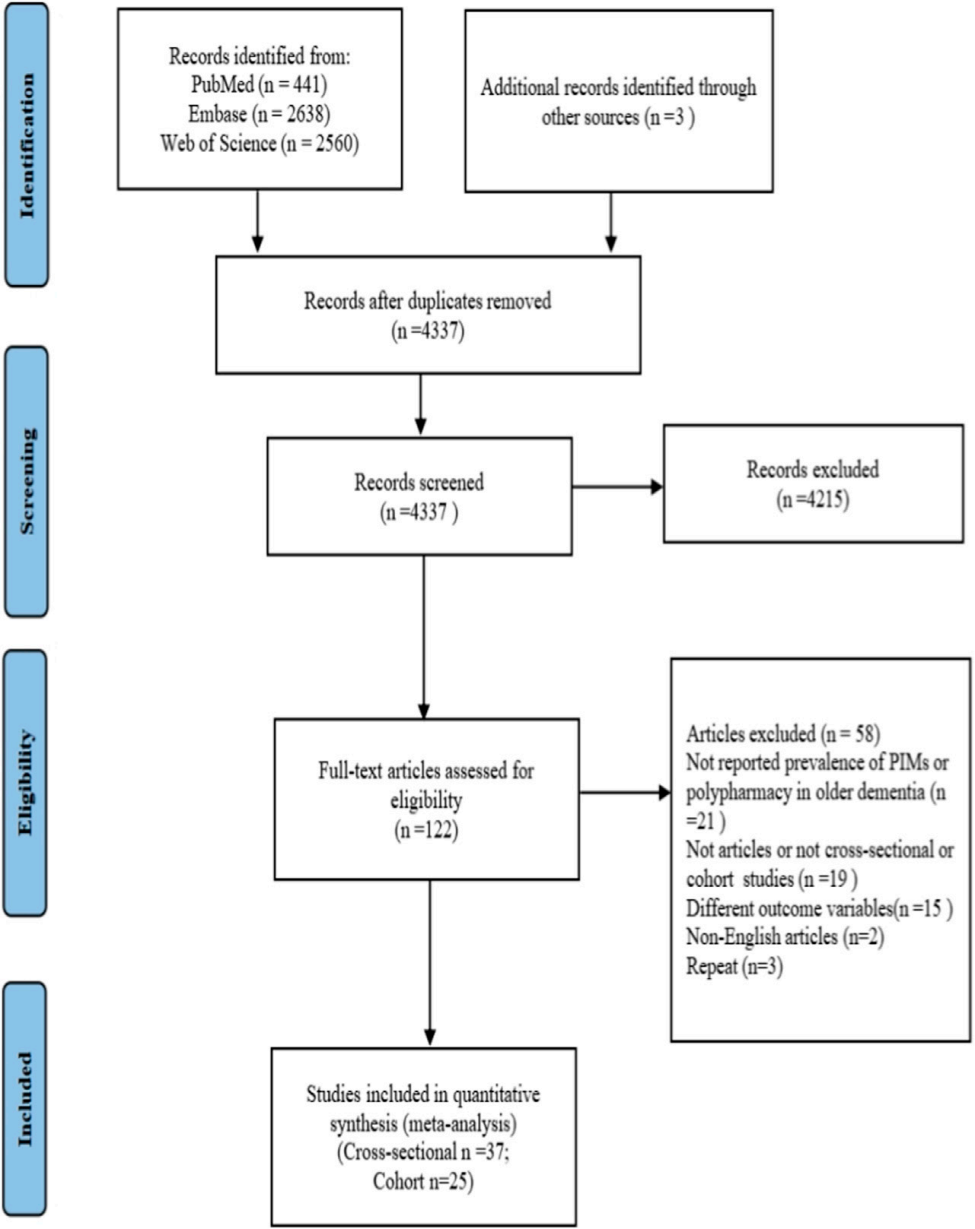
PRISMA flow diagram. PRISMA, Preferred Reporting Items for Systematic Reviews and Meta-Analyses; PIM, potentially inappropriate medication.

Overall, 5,642 records were initially obtained through PubMed, Embase, and Web of Science databases. After removing duplication (n = 1,302), 4,337 records were used to screen the title and abstract. Finally, 62 studies (Zuckerman et al., 2005; Raivio et al., 2006; Holmes et al., 2008; Chan et al., 2009; Lau et al., 2010; Somers et al., 2010; Tjia et al., 2010; Andersen et al., 2011; Lau et al., 2011; Bosboom et al., 2012; Colloca et al., 2012; Parsons et al., 2012; Thorpe et al., 2012; Fiss et al., 2013; Koyama et al., 2013; Montastruc et al., 2013; Toscani et al., 2013; Tjia et al., 2014; Hanlon et al., 2015; Skoldunger et al., 2015; Barry et al., 2016; Cross et al., 2016; Walsh et al., 2016; Chuang et al., 2017; Clague et al., 2017; Hyttinen et al., 2017; Kanagaratnam et al., 2017; Oesterhus et al., 2017; Ramsey et al., 2017; Wucherer et al., 2017; Kristensen et al., 2018; Nguyen et al., 2018; Renom-Guiteras et al., 2018; Bala et al., 2019; Brimelow et al., 2019; Denholm et al., 2019; Eshetie et al., 2019a; Kristensen et al., 2019; Soysal et al., 2019; Eshetie et al., 2020; Eshetie et al., 2020; Forgerini et al., 2020; Murphy et al., 2020; Rausch and Hoffmann, 2020; Ruangritchankul et al., 2020; Delgado et al., 2021; Ferreira et al., 2021; Gareri et al., 2021; Growdon et al., 2021; Jaramillo-Hidalgo et al., 2021; Kristensen et al., 2021; Thapaliya et al., 2021; Vickers et al., 2021; Buckley et al., 2022; Chao et al., 2022; Delgado et al., 2022; Rangfast et al., 2022; Riedl et al., 2022; Yoon et al., 2022; Zhao et al., 2022; Bae-Shaaw et al., 2023; Ryskina et al., 2023) were included based on the eligibility criteria after thoroughly reading the full text (n = 122). The flow diagram of literature screening is shown in Figure 1.

### 3.2 Characteristics of included studies

Of all included studies, 53 studies reported the prevalence of PIM (Zuckerman et al., 2005; Raivio et al., 2006; Holmes et al., 2008; Chan et al., 2009; Lau et al., 2010; Somers et al., 2010; Tjia et al., 2010; Andersen et al., 2011; Lau et al., 2011; Bosboom et al., 2012; Colloca et al., 2012; Parsons et al., 2012; Thorpe et al., 2012; Fiss et al., 2013; Koyama et al., 2013; Montastruc et al., 2013; Toscani et al., 2013; Tjia et al., 2014; Hanlon et al., 2015; Skoldunger et al., 2015; Barry et al., 2016; Cross et al., 2016; Chuang et al., 2017; Hyttinen et al., 2017; Oesterhus et al., 2017; Ramsey et al., 2017; Wucherer et al., 2017; Kristensen et al., 2018; Nguyen et al., 2018; Renom-Guiteras et al., 2018; Eshetie et al., 2019a; Bala et al., 2019; Brimelow et al., 2019; Denholm et al., 2019; Eshetie et al., 2020; Eshetie et al., 2020; Forgerini et al., 2020; Murphy et al., 2020; Rausch and Hoffmann, 2020; Ruangritchankul et al., 2020; Delgado et al., 2021; Ferreira et al., 2021; Jaramillo-Hidalgo et al., 2021; Kristensen et al., 2021; Vickers et al., 2021; Buckley et al., 2022; Delgado et al., 2022; Rangfast et al., 2022; Riedl et al., 2022; Yoon et al., 2022; Zhao et al., 2022; Bae-Shaaw et al., 2023; Ryskina et al., 2023), and 28 studies reported the prevalence of polypharmacy (Lau et al., 2010; Somers et al., 2010; Lau et al., 2011; Bosboom et al., 2012; Montastruc et al., 2013; Hanlon et al., 2015; Cross et al., 2016; Walsh et al., 2016; Clague et al., 2017; Kanagaratnam et al., 2017; Oesterhus et al., 2017; Kristensen et al., 2018; Nguyen et al., 2018; Kristensen et al., 2019; Soysal et al., 2019; Forgerini et al., 2020; Rausch and Hoffmann, 2020; Ruangritchankul et al., 2020; Ferreira et al., 2021; Gareri et al., 2021; Growdon et al., 2021; Jaramillo-Hidalgo et al., 2021; Thapaliya et al., 2021; Vickers et al., 2021; Chao et al., 2022; Delgado et al., 2022; Riedl et al., 2022; Zhao et al., 2022). The sample size ranged from 34 to 259,291, comprising a total of 658,431 study subjects. Most studies (n = 30) were conducted in Europe (Raivio et al., 2006; Andersen et al., 2011; Colloca et al., 2012; Parsons et al., 2012; Fiss et al., 2013; Montastruc et al., 2013; Toscani et al., 2013; Skoldunger et al., 2015; Barry et al., 2016; Hyttinen et al., 2016; Walsh et al., 2016; Clague et al., 2017; Kanagaratnam et al., 2017; Oesterhus et al., 2017; Wucherer et al., 2017; Kristensen et al., 2018; Renom-Guiteras et al., 2018; Denholm et al., 2019; Kristensen et al., 2019; Soysal et al., 2019; Murphy et al., 2020; Rausch and Hoffmann, 2020; Delgado et al., 2021; Gareri et al., 2021; Jaramillo-Hidalgo et al., 2021; Kristensen et al., 2021; Buckley et al., 2022; Delgado et al., 2022; Rangfast et al., 2022; Riedl et al., 2022), only two studies were conducted in South America (Forgerini et al., 2020; Ferreira et al., 2021), and the rest of the studies conducted in Oceania (Somers et al., 2010; Bosboom et al., 2012; Cross et al., 2016; Bala et al., 2019; Eshetie et al., 2019b; Brimelow et al., 2019; Eshetie et al., 2020; Eshetie et al., 2020; Ruangritchankul et al., 2020; Thapaliya et al., 2021) (*n* = 10), North America (Zuckerman et al., 2005; Holmes et al., 2008; Chan et al., 2009; Lau et al., 2010; Tjia et al., 2010; Lau et al., 2011; Thorpe et al., 2012; Koyama et al., 2013; Tjia et al., 2014; Hanlon et al., 2015; Ramsey et al., 2017; Growdon et al., 2021; Vickers et al., 2021; Bae-Shaaw et al., 2023; Ryskina et al., 2023) (*n* = 15), and Asia (Chuang et al., 2017; Nguyen et al., 2018; Chao et al., 2022; Yoon et al., 2022; Zhao et al., 2022) (*n* = 5). Table 1 presents the characteristics of the included studies.

**TABLE 1:** Characteristics of included studies.

Study	Year	Setting	Country	Region	Type	Criteria	Age (mean or median)	Female (%)	Sample	PIM	PP	Identification of dementia population
Andersen et al	2011	Community	Norway	Europe	Cross-sectional	STOPP v1	80.9 (±7.1)	60.0%	187	37.00%	NR	ICD-10
Bala et al	2019	Community	New Zealand	Oceania	Cross-sectional	2015 Beers Criteria	NR*	NR	2,190	66.90%	NR	CPS
Barry et al	2016	Community	Ireland	Europe	Cross-sectional	STOPP (36) v2	79.6 (±8.0)	64.4%	6,826	64.40%	NR	Anti-dementia drugs
Bosboom et al	2012	Care homes	Australia	Oceania	Cross-sectional	2003 Beers criteria	85.9 (±7.7)	74.8%	226	54.90%	92.00%	Clinical diagnosis of dementia and MMSE<=24
Brimelow et al	2012	Care homes	Australia	Oceania	Cross-sectional	2012 Beers criteria	86 (±8.9) a	74.3%a	441	50.40%	NR	Medical records
Chao et al	2022	Inpatient	China	Asia	Cross-sectional	-	86 (79–90)	37.8%	74	-	79.70%	NIA—AA or DSM-5
Chan et al	2008	Community	United States	North America	Cross-sectional	2003 Beers criteria	81.5 (±6.2)	78.0%	118	82.20%	NR	DSM-IV
Clague et al	2017	Community	UK	Europe	Cross-sectional	-	82.6 (±7.4)	70.6%	10,528	-	57.11%	Standard clinical coding system in use in the UK primary care
Colloca et al	2012	Outpatient	Italy, France, Finland	Europe	Cross-sectional	Holmes criteria	84.2 (±8.9)	75.0%	1,449	44.90%	NR	CPS
Cross et al	2016	Community	Australia	Oceania	Cross-sectional	2012 Beers/STOPP v2	77.6 (±7.4) a	NA	779	21.05%	68.30%	DSM-IV
Ferreira et al	2021	Community/care homes	Brazil	South America	Cross-sectional	2019 Beers criteria	NR*	65.0%	234	66.70%	45.30%	ICD-10
Fiss et al	2011	Outpatient	Germany	Europe	Cross-sectional	PRISCUS list	82.7 (±6.8)	31.7%	111	27.00%	NR	DemTect score
Forgerini et al	2020	Community	Brazil	South America	Cross-sectional	Modify PIM list	81 (76–87)	67.1%	143	63.60%	57.30%	ICD-10
Gareri et al	2020	Community	Italy	Europe	Cross-sectional	2019 Beers criteria	82.4 (±8.4)	64.8%	972	-	85.20%	Medical records
Growdon et al	2021	Outpatient	United States	North America	Cross-sectional	-	81	63.0%	918	-	72.00%	ICD-9 and ICD-10
Hanlon et al	2015	Care homes	United States	North America	Cross-sectional	2012 Beer criteria	NR*	3.0%	1,303	26.90%	26.25%	ICD-9
Hidalgo et al	2021	Community	Spain	Europe	Cross-sectional	STOPP Frail criteria	89 (87–93)	76.0%	100	85.00%	81.00%	FAST
Holmes et al	2008	Community/care homes	United States	North America	Cross-sectional	Holmes criteria	83.8	74.0%	34	29.00%	NR	FAST
Kristensen et al	2018	Community/care homes	Denmark	Europe	Cross-sectional	Red–yellow–green list	83.2 (77.5–88.2)	64.0%	35,476	24.40%	62.60%	ICD-10
Kristensen et al	2019	Community/care homes	Denmark	Europe	Cross-sectional	-	83.0 (77.3–88.0)	63.8%	33,870	-	68.10%	ICD-10
Kristensen et al	2020	Community/care homes	Denmark	Europe	Cross-sectional	Red–yellow–green list	NR*	63.3%	36,031	43.50%	NR	ICD-10
Lau et al	2010	Community	United States	North America	Cross-sectional	2003 Beers criteria	77.8 (±6.8)	53.2%	2,467	15.00%	51.92%	CDR global score and Functional Activities Questionnaire total score
Montastruc et al	2013	Community	France	Europe	Cross-sectional	Beers criteria/Laroche list	77.9 (±6.8)	71.1%	684	Beer 25.3%	43.70%	DSM-IV and NINCDS-ADRDA criteria
										Laroche 46.8%		
Oesterhus et al	2017	Community	Norway	Europe	Cross-sectional	NORGEP criteria	77 (71–81)	58.0%	251	14.00%	45.00%	DSM-IV
Parsons et al	2012	Care homes	UK	Europe	Cross-sectional	STOPP v1 (31)	86.8 (±6.7)	79.8%	119	46.20%	NR	ICD-10
Rangfast et al	2022	Community	Sweden	Europe	Cross-sectional	Sweden and national welfare	82.7 (±6.6)	61.8%	35,212	21.70%	NR	ICD-10
Riedl et al	2022	Community/care homes	Germany	European	Cross-sectional	2019 Beers criteria	74.1 (±11.1)	56.0%	191	39.00%	49.70%	ICD-9
Ruangritchanku et al	2020	Community/care homes	Australia	Oceania	Cross-sectional	2019 Beers criteria	82.3 (±7.1)	53.8%	416	56.00%	78.00%	Medical records and CPS
Somers et al	2010	Care homes	Australia	Oceania	Cross-sectional	2003 Beers criteria	85.2 (±7.8)	75.0%	351	50.40%	91.00%	Medical records
Thorpe et al	2012	Community	United States	North America	Cross-sectional	2003 Beers criteria	79.5 (±6.6)	39.1%	566	33.00%	NR	Medical records
Tjia et al	2014	Care homes	United States	North America	Cross-sectional	Holmes criteria	NR*	78.4%	5,406	53.90%	NR	Medical records and CPS
Nguyen et al	2018	Outpatient	Vietnam	Asia	Cross-sectional	Vietnamese PIMcog list	71.9 (±11.0)	51.6%	128	41.40%	14.10%	Medical records
Vickers et al	2021	Community	United States	North America	Cross-sectional	2015 Beers Criteria	NR*	60.1%	73	33.20%	58.10%	ICD-10
Walsh et al	2016	Outpatient	Ireland	Europe	Cross-sectional	-	84 (79–89)	57.7%	147	-	83.70%	Medical records
Wucherer et al	2017	Community	Germany	Europe	Cross-sectional	PRISCUS list	NR*	NR	168	16.10%	NR	ICD-10
Yoon et al	2020	Outpatient	Korea	Asia	Cross-sectional	2015 Beer criteria	77 (73–81)	63.1%	2,100	47.00%	NR	ICD-10
Zhao et al	2022	Outpatient	China	Asia	Cross-sectional	2019 Beer criteria	80.88 (±7.69)	49.2%	18,624	39.43%	16.15%	ICD-10
Bae-Shaaw et al	2023	Community	United States	North America	Cohort	2019 Beers criteria	81.4 (±8.1)	64.5%	259,291	31.63%	NA	ICD-9 or ICD-10
Buckley et al	2022	Inpatient	Ireland	European	Cohort	2015 Beers or STOPP criteria v2	78 (73–83) a	NA	261	29.90%	NA	DSM-IV
Chuang et al	2017	Inpatient/outpatient	China	Asia	Cohort	Holmes criteria	85 (80–89)	46.9%	6,532	10.47%	NA	ICD-9
Delgado et al	2020	Community/nursing home	UK	European	Cohort	STOPP v2	84.4 (±7.4)	65.6%	11,175	73.50%	NA	Medical records
Delgado et al	2022	Primary/second care	UK	European	Cohort	STOPP v2	84.5 (±7.4)	65.7%	9,324	75.4	81.60%	Medical records
Denholm et al	2019	Primary care	UK	European	Cohort	Holmes criteria	86.6 (±7.3)	64.0%	6,923	49.90%	NA	ICD-10
Eshetie et al	2019	Community	Australia	Oceania	Cohort	STOPP v2	80 (75–85)	60.0%	1,176	85%	NA	Anti-dementia drugs
Eshetie et al	2020	Community	Australia	Oceania	Cohort	STOPP v2	83 (77–88)	63.4%	8,280	79.10%	NA	Anti-dementia drugs
Eshetie et al	2020	Inpatient	Australia	Oceania	Cohort	2019 Beers criteria	87 (81.7–91)	51.7%	91	84.60%	NA	Medical records
Hyttinen et al	2016	Community	Finland	European	Cohort	Finnish criteria	NR*	64.7%	50,494	12.20%	NA	Medical records
Kanagaratnam et al	2017	Inpatient	France	European	Cohort	-	82 (±8)	61.4%	293		83.60%	DSM-IV
Koyama et al	2013	Community	United States	North America	Cohort	2003 Beers criteria	NR*	100.0%	260	33.10%	NA	DSM-IV
Lau et al	2011	Community	United States	North America	Cohort	2003 Beers criteria	77.4 (±6.6)	49.2%	1994	16.20%	48.70%	CDR
Murphy et al	2020	Community	Ireland	European	Cohort	STOPP v2	72.56 (±8.19)	62.3%	448	55.80%	NA	NINCDS-ADRDA criteria combined with MMSE
Raivio et al	2006	Inpatient/nursing home	Finland	European	Cohort	2003 Beers criteria	86a	85.5%	255	36.90%	NA	DSM-IV
Ramsey et al	2018	Inpatient	United States	North America	Cohort	2015 Beers Criteria	80.5 (±7.8)	51.8%	2,448	63.40%	NA	Medical records
Rausch et al	2020	Nursing home	Germany	European	Cohort	Holmes criteria	86.4 (±6.5)	67.8%	29,052	26.80%	85.20%	ICD-10
Renom-Guiteras et al	2018	ILTC facility/home care	England, Estonia, Finland, France, Germany, the Netherlands, Spain, and Sweden	European	Cohort	European Union (7)-PIM list	83 (±6.6)	67.5%	2,004	60%	NA	Standard diagnosis of dementia and MMSE
Ryskina et al	2023	Nursing home	United States	North America	Cohort	2019 Beer criteria	NR*	71.10%	54,713	49.50%	NA	Validated algorithm based on medical information
Skoldunger et al	2015	Community/nursing home	Norway	European	Cohort	Swedish National Board of Health and Welfare	74.8 (±11.1) a	NA	319	27%	NA	DSM-III
Soysal et al	2019	Community	UK	European	Cohort	-	80.7 (±8.7)	61.1%	12,148	-	39%	ICD-10
Thapaliya et al	2021	Community	Australia	Oceania	Cohort	_	NR*	100.0%	970	-	67.42%	Medical records
Tjia et al	2010	Nursing home	United States	North America	Cohort	Holmes criteria	85.3 (±7.5)	85.4%	323	37.50%	NA	Medical records and CPS
Toscani et al	2013	Community/nursing home	Italy	European	Cohort	Holmes criteria	86.0 (81–92)	80.3%	410	2%	NA	Medical records and FAST
Zuckerman et al	2005	Nursing home	United States	North America	Cohort	1997 Beer criteria	NR*	NA	334	19%	NA	Diagnostic and Statistical Manual of Mental Disorders Ⅲ

NR*: reported, but inclusion criteria limited to older population aged 65 years or above.

^a^ represents the total sample; PIM, potentially inappropriate medication; pp, polypharmacy; FAST, Functional Assessment Staging Tool.

MMSE, mini-mental state examination; CPS, Cognitive Performance Scale; DSM, the diagnostic and statistical manual of mental disorders; NINCDS-ADRDA, National Institute of Neurological and Communicative Disorders and Stroke/Alzheimer’s Disease; CDR, the Clinical Dementia Rating; NIA—AA, National Institute on Aging—Alzheimer’s Association.

### 3.3 Quality of the included studies

The results of quality assessment are presented in Supplementary Table S2, 3. For the cross-sectional study, we found that the lowest score was 4, and the highest score was 8. Seven research articles were of high methodological quality (AHRQ score ≥8), and 30 articles were of moderate methodological quality (AHRQ score 3–8). For the cohort studies, 20 research articles were of high methodological quality (NOS score ≥8), four articles were of moderate methodological quality (NOS score 6–7), and one article was of low quality (NOS score ≤5).

### 3.4 Prevalence of polypharmacy

**FIGURE 2:**
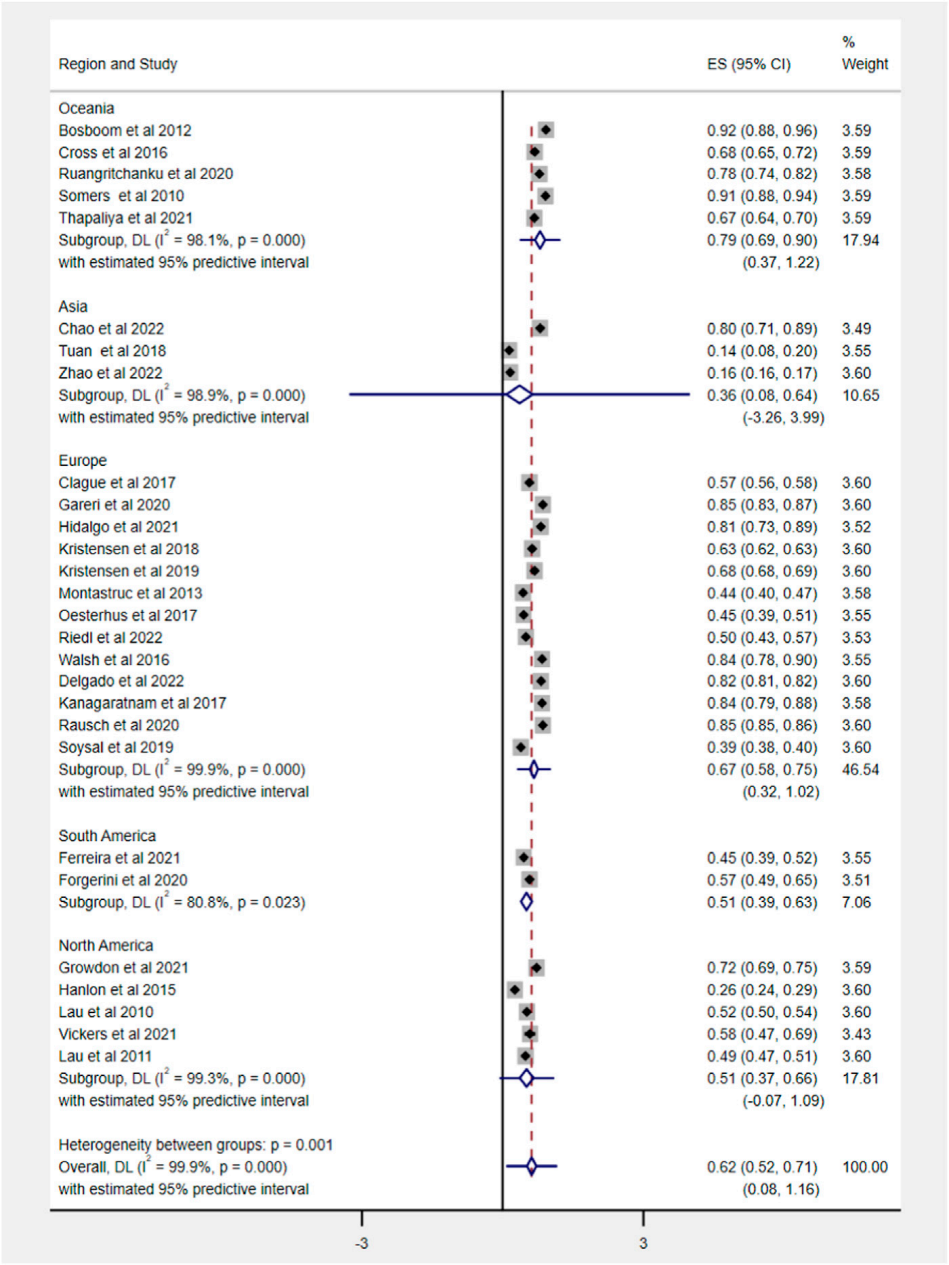
Prevalence of polypharmacy in older people with dementia across various geographic regions. Note that with <3 studies, the distribution is inestimable and hence not displayed.

Out of 62 included studies, 28 studies, comprising 4,813,226 older patients with dementia, reported the prevalence of polypharmacy, ranging from 14.10% to 92%. The pooled estimate of polypharmacy was 62% (95% CI 52–71). After weighing the population size by region, a significant difference among different regions was observed (df = 4, *p* < 0.0001). The pooled prevalence in Oceania was highest (79%, 95% CI 69–90) and lowest in Asia (36%, 95% CI 8–64). The detailed data about regions are shown in Figure 2.

### 3.5 Prevalence of potentially inappropriate medication

**FIGURE 3:**
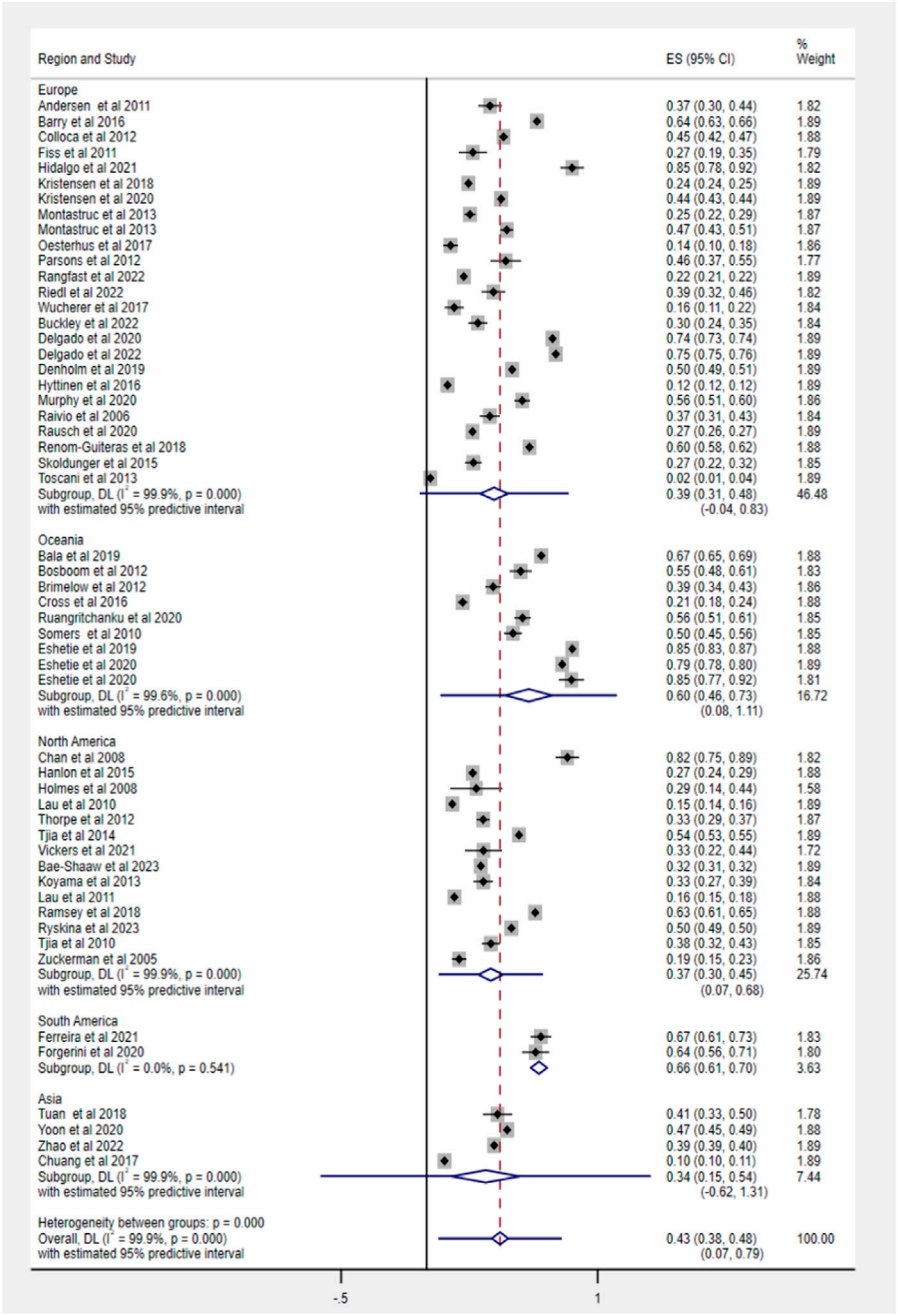
Prevalence of PIM use in older people with dementia across various geographic regions.

Fifty-three studies evaluated the prevalence of PIM based on different criteria, ranging from 2% to 85.1%. The pooled prevalence estimate of PIM was 43% (95% CI 38–48), as shown in Figure 3. Among regions, the prevalence of PIM showed a significant difference in statistics (Q = 59.5, df = 4, *p* < 0.0001), ranging from 34% in Asia (95% CI 15–54) to 66% in South America (95% CI 61–70).

### 3.6 Stratified analysis

We applied the stratified analysis for estimating the substantial heterogeneity in pooled prevalence of polypharmacy and PIM. Based on various basic characteristics, such as the proportion of females, year of publication, study design, and severity of dementia, we stratified the studies.

The subgroup analysis based on study design found no significant heterogeneity between groups in PIM use (cross-sectional: 42%, 95% CI 37–48; cohort: 44%, 95% CI 35–52). According to PIM criteria, we estimated that the pooled prevalence of PIM using the STOPP tool was highest (68%, 95% CI 63–73), following Beers criteria (44%, 95% CI 39–49) and other screening tools (32%, 95% CI 26–38). The specific data information in each subgroup for PIM use and polypharmacy is summarized in Table 2.

**TABLE 2:** Stratified meta-analysis of the prevalence of polypharmacy and PIM use.

Characteristics	Number of studies	Pooled prevalence (95% CI)	95% PI	I2 (%)	Z	Heterogeneity between groups		
						Q	df	P
PIM								
Year of publication						5.15	1	0.023
≤2015	21^a^	0.36 (0.27, 0.44)	(-0.08, 0.79)	99.5%	8.15			
>2015	32	0.48 (0.42, 0.54)	(0.07,0.79)	100%	15.12			
Percentage of female						6.98	1	0.008
<50%	6	0.26 (0.12, 0.40)	(-0.27, 0.78)	99.8%	3.57	9.88	2	0.007
≥50%	40^a^	0.47 (0.42, 0.53)	(0.11, 0.84)	99.9%	16.68			
NR	7	0.31 (0.13, 0.50)	(-0.38,1.00)	99.4%	3.28			
Mean age						5.75	2	0.056
≥80	27	0.50 (0.43, 0.57)	(0.12, 0.87)	99.9%	14.25			
<80	10^a^	0.36 (0.22, 0.51)	(-0.14, 0.86)	99.8%	4.89			
NR	16	0.36 (0.25, 0.46)	(-0.22, 0.94)	99.9%	6.35			
Criteria						98.57	2	<0.0001
Beers	23	0.44 (0.39, 0.49)	(0.17, 0.70)	99.8%	16.42			
STOPP	9	0.68 (0.63, 0.73)	(0.51, 0.85)	98.9%	28.04			
Other	21^a^	0.32 (0.26, 0.38)	(0.03, 0.61)	99.9%	10.87	--	--	--
Degree of dementia						79.60	3	<0.0001
Mild	1	0.14 (0.10, 0.18)	-	-	6.40			
Mild–moderate	3^a^	0.39 (0.25, 0.52)	(-0.27, 1.04)	98.4%	5.62			
Advanced	10^a^	0.36 (0.25, 0.45)	(-0.07, 0.80)	99.9%	6.29			
NR	40	0.45 (0.40, 0.50)	(0.07, 0.82)	99.9%	15.51			
Type						0.06	1	0.8
Cross-sectional	31^a^	0.42 (0.37, 0.48)	(0.12, 0.73)	99.8%	16.13			
Cohort	22	0.44 (0.35, 0.52)	(0.00, 0.79)	100%	10.05			
Polypharmacy								
Year of publication						0.11	1	0.74
≤2015	6	0.59 (0.40, 0.78)	(-0.13, 1.31)	99.7%	6.00			
>2015	22	0.63 (0.52, 0.74)	(0.07, 1.18)	100.0%	11.27			
Percentage of female						7.71	2	0.021
<50%	4	0.42 (0.23, 0.61)	(-0.49, 1.34)	99.7%	4.41			
≥50%	23	0.65 (0.59, 0.71)	(0.32, 0.98)	99.8%	19.93			
NR	1	0.68 (0.65, 0.72)	-	-	40.97			
Mean age						15.09	2	0.001
≥80	17	0.71 (0.59, 0.84)	(0.14, 0.29)	100%	11.22			
<80	6	0.42 (0.35, 0.50)	0.15, 0.70)	96.5%	10.85			
NR	5	0.53 (0.32, 0.74)	0.62 (0.08, 1.16)	99.4%	4.92			
Type						0.46	1	0.496
Cross-sectional	22	0.60 (0.50, 0.71)	(0.06, 1.15)	99.9%	11.05			
Cohort	6	0.68 (0.50, 0.86)	(0.00, 1.35)	99.9%	7.33			

PIM, potentially inappropriate medication; PI, prediction interval.

^a^ One study reported two PIM prevalence using different PIM lists.

Note that with <3 studies, the distribution is inestimable and hence not displayed.

### 3.7 Factors associated with potentially inappropriate medications

**FIGURE 4:**
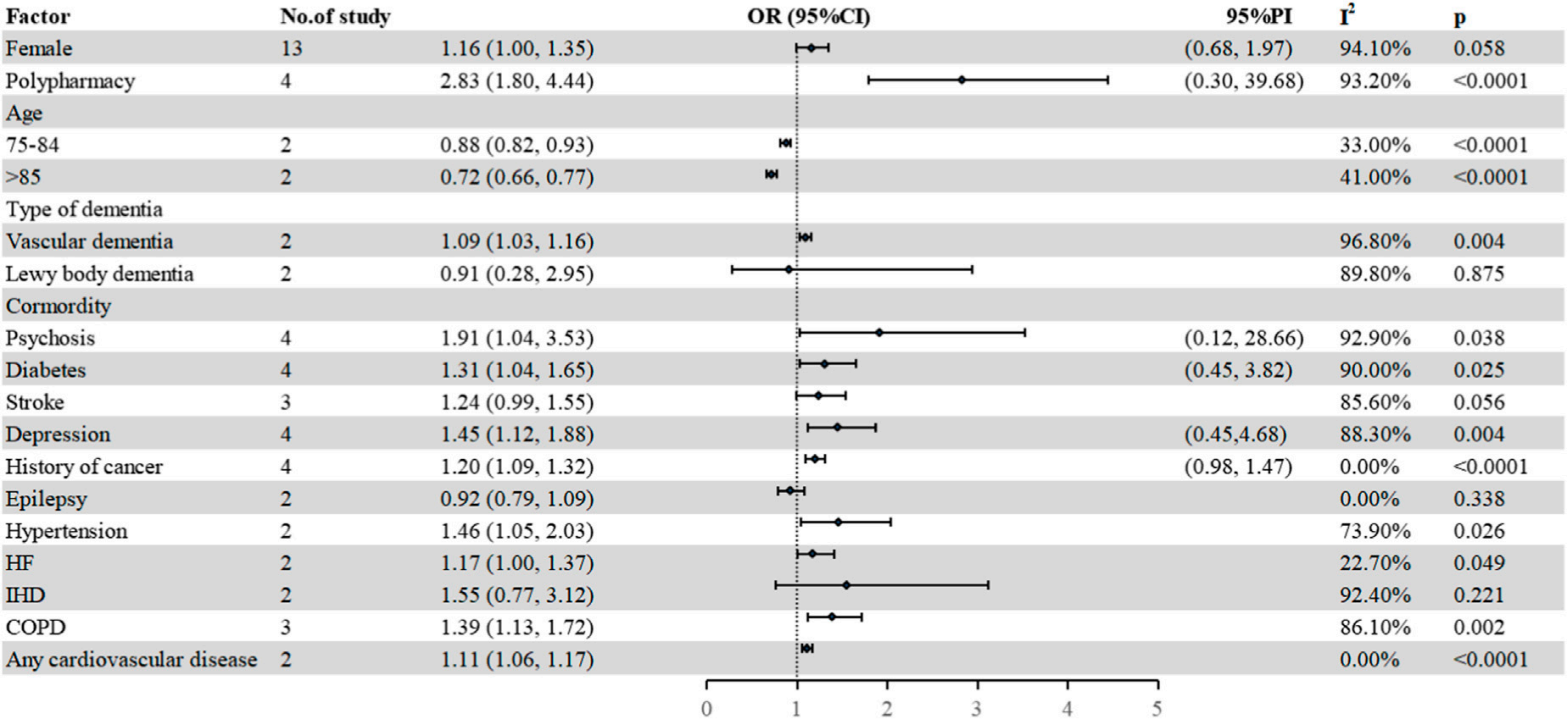
Factors associated with PIM use. HF, heart failure; IHD, ischemic heart disease; COPD, chronic obstructive pulmonary disease; PI, prediction interval.

A total of sixteen studies (Lau et al., 2010; Colloca et al., 2012; Fiss et al., 2013; Montastruc et al., 2013; Barry et al., 2016; Chuang et al., 2017; Hyttinen et al., 2017; Oesterhus et al., 2017; Renom-Guiteras et al., 2018; Murphy et al., 2020; Delgado et al., 2021; Ferreira et al., 2021; Rangfast et al., 2022; Tuan et al., 2018; Yoon et al., 2022; Zhao et al., 2022) referred to potential confounding factors with PIM in older patients with dementia. The specific details of each study reporting factors are shown in Supplementary Table S4. In the study, 15 factors, namely, age, female, polypharmacy, type of dementia, diabetes, heart failure, depression, psychosis, epilepsy, hypertension, stoke, ischemic heart disease, history of cancer, and chronic obstructive pulmonary disease or asthma were further analyzed (Figure 4).

Four studies (Montastruc et al., 2013; Ferreira et al., 2021; Yoon et al., 2022; Zhao et al., 2022) investigated the relationship between polypharmacy (five or more) and the risk of PIMs in older patients with dementia. The pooled estimate was 2.83 (95% CI 1.80–4.44), which indicated that increasing PIM risk was related with polypharmacy.

Regarding gender, a total of thirteen studies (Colloca et al., 2012; Fiss et al., 2013; Montastruc et al., 2013; Barry et al., 2016; Chuang et al., 2017; Hyttinen et al., 2017; Oesterhus et al., 2017; Nguyen et al., 2018; Murphy et al., 2020; Ferreira et al., 2021; Rangfast et al., 2022; Yoon et al., 2022; Zhao et al., 2022) were pooled to explore the association between females and PIM in older patients with dementia, in which six studies (Fiss et al., 2013; Montastruc et al., 2013; Barry et al., 2016; Oesterhus et al., 2017; Yoon et al., 2022; Zhao et al., 2022) showed that women were positively associated with PIM use, six studies (Colloca et al., 2012; Chuang et al., 2017; Murphy et al., 2020; Ferreira et al., 2021; Tuan et al., 2018; Rangfast et al., 2022) showed no correlation in statistics, and one study showed a negative association with PIM use (Hyttinen et al., 2017). The pooled estimate was 1.16 (95% CI 1.00–1.35). Three studies (Lau et al., 2010; Rangfast et al., 2022; Tuan et al., 2018) mentioned the impact of the type of dementia on PIM use. Two studies (Rangfast et al., 2022; Tuan et al., 2018) were further pooled to explore the relationship between the type of dementia and PIM use. Compared with Alzheimer’s disease (AD), patients with vascular dementia were more likely to suffer PIM, while in case of Lewy body dementia, there was no difference in statistics (vascular dementia: 1.09, 95% CI 1.03–1.16; Lewy body dementia: 0.91, 95% CI 0.28–2.95). For other potential confounding factors (age, diabetes, hypertension, psychosis, and heart failure), the details are shown in Figure 4.

### 3.8 Publication bias assessment

Egger’s and Begg’s tests were used to assess the publication bias in the pooled estimate of polypharmacy and PIM. The results for the pooled estimate of polypharmacy were 0.855 (Egger’s test) and 0.767 (Begg’s test), indicating no publication bias. Regarding PIM, no statistically significant publication bias was observed (Egger test: *p* = 0.058; Begg’s test: *p* = 0.765).

## 4 Discussion

To our knowledge, the current study first comprehensively summarized the pooled prevalence of PIM and polypharmacy in older patients with dementia across different regions and analyzed potential confounding factors associated with PIM use. This review may provide evidence for healthcare decision-makers in avoiding adverse drug use events in elderly with dementia.

For the included studies, the prevalence of PIM varied widely, ranging from 2% to 85.1%. Several reasons could explain the phenomenon well. First, the difference in marketing drugs and medical habits and the gap in healthcare systems in different regions might significantly affect the prevalence of PIM. Based on the pooled results by region, we clearly found a vast difference in PIM prevalence. Renom-Guiteras et al. evaluated the PIM prevalence of eight European countries using the European Union (7)-PIM list and found the PIM prevalence ranging 47%–67.5% (Renom-Guiteras et al., 2018). Zhao et al. also reported differences in PIM prevalence across different cities in China, ranging from 28.48% to 44.79% (Zhao et al., 2022). Second, the screening tools were considered a factor resulting in differences in PIM. To date, many screening tools have been applied to evaluate PIM, such as Beers criteria, STOPP/START criteria, and Holmes criteria (American Geriatrics Society Beers Criteria^®^ Update Expert Panel, 2019; O'Mahony et al., 2018; Holmes et al., 2008). In our study, a total of 13 criteria were used, of which the most frequently used was Beers criteria, accounting for nearly 50%, followed by STOPP/START criteria. Published PIM lists have important differences in terms of contents and number (e.g., 81 items in STOPP criteria, while 91 in Beers criteria), which might lead to different prevalence of PIM. Target population and clinical practice among various PIM lists might also affect PIM prevalence, such as Holmes criteria mainly focusing on advanced dementia and NORGEP criteria for ambulatory patients (Kaufmann et al., 2014). The stratified analysis based on different criteria in our study also found differences in PIM prevalence. Although the same tool was used, the proportion of patients receiving PIM still varied, which were mainly attributed to how the tools were applied and the edition of the criteria. For instance, several studies just used part of the items of Beers criteria due to the absence of diagnostic information or other laboratory indicators, underestimating PIM to some extent (Chan et al., 2009; Lau et al., 2010; Thorpe et al., 2012; Montastruc et al., 2013). Therefore, before conducting research, researchers must consider how to select appropriate tools and how to apply them based on the collected information and diagnoses. In addition to the impact of region and screening tools on PIM, severity of dementia should be considered. In the analysis, we found that advanced dementia has a lower pooled estimate of PIM than mild–moderate dementia. Despite that patients living with advanced dementia depended completely on others, suffering from a series of distressing symptoms, such as neuropsychiatric symptoms and pain (Moens et al., 2014; Hendriks et al., 2015; Sampson et al., 2018), the emphasis of therapy for those was on ensuring patient comfort and symptom management and reducing polypharmacy (Disalvo et al., 2016). A review by Parsons summarized a viewpoint of physicians about drug use for advanced dementia, recommending discontinuation of anticholinesterase inhibitors, memantine, quetiapine, and simvastatin (Parsons et al., 2010). This may lead to a lower prevalence of PIM in advanced dementia. Despite the vast difference in PIM prevalence among included studies, the pooled estimate of PIM use in our analysis was up to 43%, which was higher than the PIM estimate for older patients in worldwide as given in Tian et al. (2023). Thus, we should pay great attention on the PIM use of older patients with dementia.

The drug management of older patients living with dementia often takes place in the context of additional comorbidities, which result in a large number of prescriptions for patients with dementia (Blass et al., 2008; Callahan and Schubert, 2014; Amy et al., 2018). In the analysis, polypharmacy was found to be prevalent with an estimated overall prevalence of 62%, slightly higher than that found in the study by Janice et al., with an estimate of 59% (Toh et al., 2023). Overall, significant heterogeneity was observed in the prevalence of polypharmacy. The difference may be attributed to several factors, for example, study subjects from different settings, geographical regions, study design, and year of publication. Although heterogeneity did not decrease by subgroups, significant differences were observed between some groups. In our review, we clearly found that the prevalence of polypharmacy in Asia was lowest compared with other regions. This may be due to socioeconomic-related healthcare inequalities between developing and developed countries in the access to healthcare. A report from the WHO declared that developed regions account for 11.6% of the worldwide burden, but account for 90.2% of health expenditure worldwide (Murray and Lopez, 1997). Different settings might affect the prevalence of polypharmacy. In this study, two out of three studies in the Asian region were outpatient studies, with a proportion of only over 10% for polypharmacy. Of note, the same definition of polypharmacy (five or more) shows a difference in measurement, which also might affect the outcome. Lau et al. declared that medications for topical applications, vitamins, and herbal medications were excluded in polypharmacy, with the prevalence of 51.92%, while Bosboom et al. considered all different medications in prescription were being counted, with the prevalence of 92% (Lau et al., 2011; Bosboom et al., 2012). In addition, the time of exposure to the medications has an impact on polypharmacy estimates. Kristensen et al. and Rausch et al. reported the prevalence of polypharmacy at 3 and 6 months as 62.6% and 85.2%, respectively (Kristensen et al., 2018; Rausch and Hoffmann, 2020). Although differences in distribution of regions, measurement, and exposure of time affect the estimate of polypharmacy, polypharmacy still cannot be ignored.

Several studies have been reported regarding potential confounding factors associated with PIM use. The association between PIM and polypharmacy for older patients with dementia was always discussed by researchers. Due to different definitions of polypharmacy and other factors, different studies concluded different results. Ferreira et al. reported polypharmacy was not related to PIM use (Ferreira et al., 2021), while Yoon et al. declared that a strong association between polypharmacy and PIM use was observed (Yoon et al., 2022). In our study, patients with polypharmacy (five or more) were exposed to a higher risk of PIM use, which was consistent with the observation of Tian et al. (2021) (reported older patients). A growing body of studies reported the impact of gender on PIM use, but a consensus has not yet been reached. According to our meta-analysis, no statistically significant difference was observed in women. Of note, we found vascular dementia was more susceptible to PIM use than Alzheimer’s disease. This may be related to cardiovascular events being the main cause of vascular dementia (Leys et al., 2005). Cardiovascular diseases and well-recognized high-risk factors of cardiovascular diseases, diabetes and hypertension, were associated with PIM use. This may be due to the use of non-steroidal drugs, regular insulin, and sulfonylureas of the PIM list. Other factors, such as comorbidity, psychosis, depression, and history of cancer, were considered to increase the risk of PIM in the current study. The long-term use of antidepressants, antipsychotics, and opioids in order to control symptoms may explain the phenomenon. Although several factors associated with PIM use were identified in our study, more relevant research was still needed for further validation.

PIM and polypharmacy in older patients with dementia are common. A large number of studies have shown PIM use and polypharmacy were related to hospitalization and death (Gnjidic et al., 2012; Maher et al., 2014). Therefore, it is necessary to optimize drug management for older patients with dementia. Deprescribing is an approach to reduce PIM and polypharmacy (Wu et al., 2021). Due to the use of multiple drugs and memory loss in older patients, drug compliance was relatively poor (Dunbar-Jacob and Mortimer-Stephens, 2001; Smith et al., 2017). For older patients with dementia featuring cognitive impairment and communication disorder, the adherence of drug medication was poorer. Deprescribing could not only reduce the number of medications taken but also increase the drug compliance of elderly patients (Basheti et al., 2016; Jäge et al., 2017). Research has confirmed the advantages of deprescribing in reducing PIM and polypharmacy (Ibrahim et al., 2021). Thus, deprescribing can be applied in the clinics to solve polypharmacy and PIM.

Although the analysis quantitatively summarized the prevalence of PIM and polypharmacy and further explored potential factors with PIM use, providing a reference for the prescription of old patients with dementia, we must acknowledge some limitations of the study. First, those studies included were from all over the world, and other factors, such as culture, education level, geographical location, and social status, would affect the results of polypharmacy and PIM. Second, the included studies show substantial heterogeneity, which may be related to the study subject, sample size, and screening tools used. Third, some studies did not specify the living conditions of the study population and type of dementia, so we cannot conduct a subgroup analysis based on living conditions and type of dementia. Fourth, included studies were limited to English articles, leading to results that could either underestimate or overestimate the prevalence. In addition, only factors using multivariate regression were extracted and several factors in our study were only examined in two studies, which gave biased results. Thus, a further study regarding factors affecting PIM use will be explored.

## 5 Conclusion

The analysis revealed that PIM use and polypharmacy were highly prevalent in older patients with dementia. Among different regions, the pooled estimate of PIM use and polypharmacy varied widely. Increasing PIM was related with polypharmacy, women, and vascular dementia. These findings highlight the necessity of some measures taken to improve the prescription quality of older patients with dementia, and they also imply that more caution should be taken when prescribing for women, polypharmacy, and vascular dementia.

## Data Availability

The original contributions presented in the study are included in the article/Supplementary Material; further inquiries can be directed to the corresponding authors.
