# Supplementary material for: Global prevalence of polypharmacy and potentially inappropriate medication in older patients with dementia: a systematic review and meta-analysis
Source: Front Pharmacol. 2023 Aug 24;14:1221069. doi: 10.3389/fphar.2023.1221069 (PMC10483131; doi:10.3389/fphar.2023.1221069)
Supplement: Supplementary file 1 [file Table1.DOCX]

Supplemental Table S1 – Search strategy

| Database | String |
| --- | --- |
| Pubmed | #1 "Inappropriate Prescribing"[Mesh]  #2 ((((Inappropriate medication[Title/Abstract] OR Inappropriate prescri*[Title/Abstract] OR OverPrescribing[Title/Abstract]) OR (Underprescribing[Title/Abstract])) OR (Misprescribing[Title/Abstract])) OR (Inappropriate drug[Title/Abstract])) OR (suboptimal prescribing[Title/Abstract])  #3 "Polypharmacy"[Mesh]  #4 "polymedication"[Title/Abstract]  # 5 Beers criteria[Title/Abstract] OR STOPP[Title/Abstract] OR STOPP START Criteria[Title/Abstract] OR FORTA Criteria[Title/Abstract] OR Holmes criteria[Title/Abstract] OR Laroche list[Title/Abstract] OR NORGEP criteria[Title/Abstract] OR PRISCUS list[Title/Abstract] OR PIM List*[Title/Abstract] OR Medication Appropriateness Index[Title/Abstract] OR Potentially Inappropriate Medication List[Title/Abstract] OR European list[Title/Abstract] OR Chinese criteria[Title/Abstract]  #6 #1 OR #2 OR #3OR #4 OR #5  #7"Dementia"[Mesh]  #8 (Amentia*[Title/Abstract] OR Familial Dementia*[Title/Abstract] OR Vascular Dementia*[Title/Abstract] OR Alzheimer Dementia*[Title/Abstract] OR Lewy Body Dementia[Title/Abstract])  #9 #7 OR #8  #10 #9 AND #6 |
| Embase | 1. exp prescribing error/   2 (Inappropriate medication or Inappropriate prescri* or Overprescribing or Underprescribing or Misprescribing).mp. [mp=title, abstract, heading word, drug trade name, original title, device manufacturer, drug manufacturer, device trade name, keyword heading word, floating subheading word, candidate term word]  3 exp polypharmacy/  4 polymedication.mp.  5 (Beers criteria or STOPP or STOPP START Criteria or FORTA Criteria or Holmes criteria or Laroche list or NORGEP criteria or PRISCUS list or PIM List* or Medication Appropriateness Index or Potentially Inappropriate Medication List or European list or Chinese criteria).mp. [mp=title, abstract, heading word, drug trade name, original title, device manufacturer, drug manufacturer, device trade name, keyword heading word, floating subheading word, candidate term word]  6 1 or 2 or 3 or 4 or 5  7 exp dementia/ or dementia.mp.  8 (Amentia* or Familial Dementia* or Vascular Dementia* or Alzheimer Dementia* or Lewy Body Dementia).mp. [mp=title, abstract, heading word, drug trade name, original title, device manufacturer, drug manufacturer, device trade name, keyword heading word, floating subheading word, candidate term word]  9 7 or 8  10 6 and 9 |
| Web of science | 1: TS=(Inappropriate medication OR Inappropriate prescri* OR Overprescribing OR Underprescribing OR Misprescribing OR suboptimal prescribing OR polypharmacy OR polymedication)  2: TS=(dementia OR Amentia* OR Familial Dementia* OR Vascular Dementia* OR Alzheimer Dementia* OR Lewy Body Dementia）  3: TS=(Beers criteria OR STOPP OR STOPP START Criteria OR FORTA Criteria OR Holmes criteria OR Laroche list OR NORGEP criteria OR PRISCUS list OR PIM List* OR Medication Appropriateness Index OR Potentially Inappropriate Medication List OR European list OR Chinese criteria)  4: #1 OR # 3  5: #4 AND #2 |

Table S2 Quality assessment of Cohort study

| S.no | (Author, year of pub.) | **Selection domain** | | | | **Comparability domain** | **Outcome domain** | | | Total Score |
| --- | --- | --- | --- | --- | --- | --- | --- | --- | --- | --- |
|  |  | Representativeness of exposed cohort | Selection of non-exposed cohort | Ascertainment of exposure | Outcome of interest was not present at start of study | Control for age or substance use or chronic medical illness; Control for other variables (2nd important variables) | Assessment of outcome | Follow-up period | Adequacy of follow up |  |
| 1 | Bae-Shaaw et al 2023 | 1 | 1 | 1 | 1 | 1 | 1 | 1 | 1 | 8 |
| 2 | Buckley et al 2022 | 1 | 1 | 1 | 1 | 2 | 1 | 1 | 1 | 9 |
| 3 | Chuang et al 2017 | 1 | 1 | 1 | 1 | 2 | 1 | 1 | 1 | 9 |
| 4 | Delgado 2020 | 1 | 1 | 1 | 1 | 2 | 1 | 1 | 1 | 9 |
| 5 | Delgado et al 2022 | 1 | 1 | 1 | 1 | 2 | 1 | 1 | 1 | 9 |
| 6 | Denholm et al 2022 | 1 | 1 | 1 | 1 | 0 | 1 | 1 | 1 | 7 |
| 7 | Eshetie 2019 | 1 | 1 | 1 | 1 | 2 | 1 | 1 | 1 | 9 |
| 8 | Eshetie 2019 | 0 | 1 | 1 | 1 | 2 | 1 | 0 | 0 | 6 |
| 9 | Eshetie 2020 | 0 | 1 | 1 | 1 | 2 | 1 | 0 | 0 | 6 |
| 10 | Hyttinen 2016 | 1 | 1 | 1 | 1 | 2 | 1 | 1 | 1 | 9 |
| 11 | Kanagaratnam et al 2017 | 1 | 1 | 1 | 1 | 2 | 1 | 0 | 1 | 8 |
| 12 | Koyama 2013 | 1 | 1 | 1 | 1 | 1 | 1 | 1 | 1 | 8 |
| 13 | Lau 2011 | 1 | 1 | 1 | 1 | 2 | 1 | 1 | 1 | 9 |
| 14 | Murphy et al 2020 | 1 | 1 | 1 | 1 | 2 | 1 | 1 | 1 | 9 |
| 15 | Raivio 2006 | 1 | 1 | 1 | 1 | 1 | 1 | 1 | 1 | 8 |
| 16 | Ramsey 2018 | 1 | 1 | 1 | 1 | 1 | 1 | 1 | 1 | 8 |
| 17 | Rausch et al 2020 | 1 | 1 | 1 | 1 | 2 | 1 | 0 | 0 | 7 |
| 18 | Renom-Guiteras et al 2018 | 1 | 1 | 1 | 1 | 2 | 1 | 1 | 1 | 9 |
| 19 | Ryskina et al 2023 | 1 | 1 | 1 | 1 | 2 | 1 | 1 | 1 | 9 |
| 20 | Skoldunger 2015 | 1 | 1 | 1 | 1 | 2 | 1 | 1 | 1 | 9 |
| 21 | Soysal et al 2019 | 1 | 1 | 1 | 1 | 1 | 1 | 1 | 1 | 8 |
| 22 | Thapaliya et al 2021 | 1 | 1 | 1 | 1 | 2 | 1 | 1 | 1 | 9 |
| 23 | Tjia 2010 | 1 | 1 | 1 | 1 | 2 | 1 | 1 | 1 | 9 |
| 24 | Toscani 2013 | 1 | 1 | 1 | 1 | 0 | 1 | 0 | 0 | 5 |
| 25 | Zuckerman 2005 | 1 | 1 | 1 | 1 | 2 | 1 | 1 | 1 | 9 |

Abbreviation:Each items in selection domain and outconme domain can have a maximum of 1 score. A maximum of two scores can be given for Comparability

Table S3 Quality assessment of Cross-sectional study

| S.no | (Author, year of pub.) | the source of data | eligible criteria for study subjects | time period for included population | whether or not subjects were consecutive | whether the outcome indicators are affected by other factors | any assessments for quality assurance | explanation for excluding any patients from analysis | measurements taken for controlling confound factors | description for the handing of missing data | summary for patient response rate and completeness of data collection | clarification of following-up results | Total Score |
| --- | --- | --- | --- | --- | --- | --- | --- | --- | --- | --- | --- | --- | --- |
| 1 | Andersen et al 2011 | 1 | 1 | 1 | 1 | 0 | 1 | 0 | 1 | 0 | 1 | 0 | 7 |
| 2 | Bala 2019 | 1 | 1 | 1 | 1 | 0 | 1 | 0 | 1 | 0 | 1 | 0 | 7 |
| 3 | Barry 2016 | 1 | 1 | 1 | 1 | 0 | 0 | 1 | 1 | 0 | 1 | 0 | 7 |
| 4 | Bosboom 2012 | 1 | 1 | 1 | 1 | 0 | 1 | 0 | 1 | 0 | 1 | 0 | 7 |
| 5 | Brimelow 2012 | 1 | 1 | 1 | 1 | 0 | 0 | 0 | 0 | 0 | 1 | 0 | 5 |
| 6 | Chan 2008 | 1 | 1 | 1 | 1 | 0 | 1 | 0 | 0 | 0 | 1 | 0 | 6 |
| 7 | Chao 2022 | 1 | 1 | 1 | 1 | 0 | 1 | 0 | 0 | 0 | 1 | 0 | 6 |
| 8 | Clague 2017 | 1 | 1 | 1 | 1 | 0 | 1 | 0 | 0 | 0 | 1 | 0 | 6 |
| 9 | Colloca 2012 | 1 | 1 | 1 | 1 | 0 | 1 | 1 | 0 | 1 | 1 | 0 | 8 |
| 10 | Cross 2016 | 1 | 1 | 1 | 1 | 0 | 1 | 0 | 1 | 0 | 1 | 0 | 7 |
| 11 | Ferreira 2021 | 1 | 1 | 1 | 1 | 0 | 0 | 1 | 1 | 0 | 1 | 0 | 7 |
| 12 | Fiss 2011 | 1 | 1 | 1 | 1 | 0 | 0 | 0 | 0 | 0 | 1 | 0 | 5 |
| 13 | Forgerini 2020 | 1 | 1 | 1 | 1 | 0 | 1 | 1 | 1 | 0 | 1 | 0 | 8 |
| 14 | Gareri 2020 | 1 | 1 | 1 | 1 | 0 | 0 | 0 | 0 | 0 | 1 | 0 | 5 |
| 15 | Growdon 2021 | 1 | 1 | 1 | 1 | 0 | 1 | 0 | 1 | 1 | 1 | 0 | 8 |
| 16 | Hanlon 2015 | 1 | 1 | 1 | 1 | 0 | 0 | 1 | 1 | 0 | 1 | 0 | 7 |
| 17 | Hidalgo 2021 | 1 | 1 | 1 | 1 | 0 | 0 | 1 | 0 | 0 | 1 | 0 | 6 |
| 18 | Homles 2008 | 1 | 1 | 1 | 1 | 0 | 0 | 0 | 0 | 0 | 1 | 0 | 5 |
| 19 | Kristensen 2018 | 1 | 1 | 1 | 1 | 0 | 0 | 1 | 1 | 0 | 1 | 0 | 7 |
| 20 | Kristensen 2019 | 1 | 1 | 1 | 1 | 0 | 0 | 1 | 1 | 0 | 1 | 0 | 7 |
| 21 | Kristensen 2020 | 1 | 1 | 1 | 1 | 0 | 0 | 0 | 1 | 0 | 1 | 0 | 6 |
| 22 | Lau 2010 | 1 | 1 | 1 | 1 | 0 | 0 | 1 | 1 | 1 | 1 | 0 | 8 |
| 23 | Montastruc 2013 | 1 | 1 | 1 | 1 | 0 | 1 | 1 | 0 | 0 | 1 | 0 | 7 |
| 24 | Oesterhus 2017 | 1 | 1 | 1 | 1 | 0 | 1 | 1 | 0 | 0 | 1 | 0 | 7 |
| 25 | Parsons 2012 | 1 | 1 | 1 | 1 | 0 | 0 | 1 | 0 | 0 | 1 | 0 | 6 |
| 26 | Rangfast 2022 | 1 | 1 | 1 | 1 | 0 | 0 | 1 | 0 | 0 | 1 | 0 | 6 |
| 27 | Riedl 2022 | 1 | 1 | 0 | 0 | 0 | 0 | 0 | 1 | 0 | 1 | 0 | 4 |
| 28 | Ruangritchanku 2020 | 1 | 1 | 1 | 1 | 0 | 0 | 0 | 0 | 0 | 1 | 0 | 5 |
| 29 | Somers 2010 | 1 | 1 | 1 | 1 | 0 | 0 | 1 | 0 | 0 | 1 | 0 | 6 |
| 30 | Thorpe 2012 | 1 | 1 | 1 | 1 | 0 | 0 | 1 | 0 | 1 | 1 | 0 | 7 |
| 31 | Tjia 2014 | 1 | 1 | 1 | 1 | 0 | 1 | 0 | 1 | 1 | 1 | 0 | 8 |
| 32 | Tuan 2018 | 1 | 1 | 1 | 1 | 0 | 0 | 1 | 1 | 1 | 1 | 0 | 8 |
| 33 | Vickers 2021 | 1 | 1 | 1 | 1 | 0 | 0 | 0 | 0 | 1 | 1 | 0 | 6 |
| 34 | Walsh 2016 | 1 | 1 | 1 | 1 | 0 | 0 | 1 | 1 | 0 | 1 | 0 | 7 |
| 35 | Wucherer 2017 | 1 | 1 | 1 | 1 | 0 | 0 | 1 | 1 | 0 | 1 | 0 | 7 |
| 36 | Yoon 2020 | 1 | 1 | 1 | 1 | 0 | 0 | 0 | 0 | 0 | 1 | 0 | 5 |
| 37 | Zhao 2022 | 1 | 1 | 1 | 1 | 0 | 1 | 1 | 0 | 1 | 1 | 0 | 8 |

Supplemental Table S4 – Factors with PIM use

| study | Variable in multiple logistic regression | adjusted risk factor （P<0.05） |
| --- | --- | --- |
| Barry 2016 | gender, age, polypharmacy | female/medication(four or more） |
| Colloca 2012 | age, Long term Stay, ADL hierarchy scale score ,Number of diseases ,Number of diseases, Diabetes, Heart failure, Stroke ,Pneumonia, Recent Hospitalization, Presence of a geriatrician，Ischemic heart disease | Heart failure, Stroke, ADL hierarchy scale score |
| Ferreira 2021 | Gender, age, polypharmacy (five or more), Time since diagnosis, Hypertension, Depression, Psychosis, Comorbidities, Level of autonomy, Type of health care | Depression, Comorbidities |
| Fiss 2011 | Gender, age, polypharmacy (reference>9), Patient receives support in drug administration, Neoplasms, Diseases of the blood and blood-forming organs and certain disorders involving the immune mechanism, Endocrine, nutritional and metabolic diseases, Mental and behavioral disorders, Diseases of the nervous system, Diseases of the respiratory system, Diseases of the digestive system, Diseases of the musculoskeletal system and connective tissue, Diseases of the genitourinary system | female; Diseases of the musculoskeletal system and connective tissue；polypharmacy(reference>9)；Patient receives support in drug administration；Endocrine, nutritional and metabolic diseases |
| Lau 2010 | Medication (ref 1 to 2), Likely cause of dementia, Severity of dementia | Medication |
| Montastruc 2013 | Gender, Polypharmacy | female； polypharmacy |
| Oesterhus 2017 | Gender, Medication (Mean), | female, medication |
| Rangfast 2022 | Gender, age, Medication (not specify), Baseline MMSE-score, Type of dementia(ref AD),Year 2017 | Vascular dementia;  Dementia with Lewy bodies and Parkinson dementia; Frontotemporal dementia; Medication |
| Tuan 2018 | age, gender, type of dementia, severity of dementia, number of comorbidities, number of medicines, number of hospital visits, and number of treating specialists. | hospital visits |
| Yoon 2020 | Gender, age, polypharmacy, Number of comorbid diseases, Parkinson’s disease, Mood (affective) disorder, Schizophrenia, Gastrointestinal disorder | female/polypharmacy/comorbid diseases/Parkinson’s disease/Mood (affective) disorder/Schizophrenia |
| Zhao 2022 | Gender, age, medication, disease, payment, total expenditure | femle/age/polypharmacy |
| Delgado 2020 | age (squared), gender, quintiles of multiple deprivation;atrial fibrillation, asthma, cancer (5 years), coronary heart disease,  chronic kidney disease (stage 3–5), chronic obstructive pulmonary disease, depression, diabetes mellitus, epilepsy, heart failure, hypertension, hypothyroidism,  severe mental illness and stroke. | Atrial fibrillation; Asthma; Coronary heart disease; Chronic kidney disease; Chronic obstructive pulmonary disease; Depression; Diabetes mellitus 2; epilepsy, heart failure, hypertension, severe mental illness and stroke; History of depression or bipolar disorders |
| Hyttinen 2016 | Age, gender, Socioeconomic position, Asthma or COPD, Diabetes, diabetes, rheumatoid arthritis, any cardiovascular disease, epilepsy and history of cancer; History of substance abuse; Opioids; psychotropic medicines; University hospital district; | Age; Female; Asthma or COPD; Any cardiovascular disease; Any cardiovascular disease; Psychotropic medicines; University hospital district |
| Renom-Guiteras 2018 | Age, Gender（ref female);Functional status; Cognitive status; Comorbidity Charlson (<=3); Setting | Age; Functional status; Comorbidity Charlson; Setting |
| Chuang 2017 | Age, Gender, Charlson Comorbidity Index(median); History of cardiovascular disease; History of diabetes mellitus; History of cancer; Hospitalizations at the end of life (n); Length of last hospital stay (days); Palliative care during last hospitalization; Tube feeding during last hospitalization | Age, History of diabetes mellitus; Length of last hospital stay (days); Hospitalizations at the end of life |
| Murphy 2020 | Age, Gender, Years of education; No.medications; No. combordity; Years since symptom onset(median);Years since diagnosis(median);Baseline CDR-sb score(median);Baseline ADAS-Cog score(median) | No. combordity; Years since symptom onset |

Table S5 List of excluded studies

| Not articles or cross-sectional or cohort studies(19) |  |  |
| --- | --- | --- |
| Alageel, N. A. | 2023 | Patterns and appropriateness of prescribing for people with dementia in ambulatory care in Saudi Arabia: A cross-sectional study |
| Coe, A. B. | 2020 | Receipt of Medicare Part D Comprehensive Medication Reviews and Potentially Inappropriate Medication Use in Older Adults with Dementia |
| Colloca, G. | 2015 | Inappropriate drug use in advanced dementia |
| Davari, P. | 2018 | Pathologic Associations of Potentially Inappropriate Medication Use in Patients with Alzheimer's Disease |
| Gareri, Pietro | 2021 | The risks of polypharmacy in ambulatory and home care patients affected with dementia: The PharE Study |
| Laroche, M. L. | 2013 | Potentially inappropriate medications in the elderly with alzheimer's disease and related dementia |
| Lassalle, M. | 2013 | Potentially inappropriate medications in the elderly with Alzheimer's disease and related dementia in France: Results of the MIDA study |
| Mehta, H. B. | 2017 | Prevalence and predictors of potentially inappropriate medications in dementia patients |
| Thorpe, J. M. | 2009 | Potentially Inappropriate Medication Use in Community-Dwelling Dementia Patients and Their Informal Caregivers |
| Gustafsson, M. | 2018 | Effects of pharmacists' interventions on inappropriate drug use and drug-related readmissions in people with dementia-a secondary analysis of a randomized controlled trial |
| Kable, Ashley | 2019 | Comparison of Potentially Inappropriate Medications for People with Dementia at Admission and Discharge during An Unplanned Admission to Hospital: Results from the SMS Dementia Study |
| Lai, S. W. | 2012 | Association between polypharmacy and dementia in older people: a population-based case-control study in Taiwan |
| Pfister, B. | 2018 | Drug-related problems and medication reviews among old people with dementia |
| Tao, P. | 2020 | Correlation between potentially inappropriate medication and Alzheimer's disease among the elderly |
| Wurm, Raphael | 2023 | Analysis of co-medication in people with dementia |
| Tjia, J. | 2015 | Inappropriate drug use in advanced dementia |
| Wilchesky, M. | 2018 | The OptimaMed intervention to reduce inappropriate medications in nursing home residents with severe dementia: results from a quasi-experimental feasibility pilot study |
| Abramsson, L. | 2020 | Prevalence of drug-related problems using STOPP/START and medication reviews in elderly patients with dementia |
| Gnjidic, D. | 2018 | The Impact of Dementia Diagnosis on Patterns of Potentially Inappropriate Medication Use Among Older Adults |
| Different outcome variables (15) |  |  |
| Bezabhe, W. M. | 2023 | Ten-Year Trends in Psychotropic Prescribing and Polypharmacy in Australian General Practice Patients with and without Dementia |
| Giron, M. S. | 2001 | The appropriateness of drug use in an older nondemented and demented population |
| Growdon, M. E. | 2022 | Attitudes toward deprescribing among older adults with dementia in the United States |
| Kachru, N. | 2015 | Potentially inappropriate anticholinergic medication use in older adults with dementia |
| Leung, A. Y. | 2013 | Residents with Alzheimer's disease in long-term care facilities in Hong Kong: patterns of hospitalization and emergency room use |
| Longo, E. | 2023 | Identifying Dynamic Patterns of Polypharmacy for Patients with Dementia from Primary Care Electronic Health Records: A Machine Learning Driven Longitudinal Study |
| Mueller, C. | 2018 | Polypharmacy in people with dementia: Associations with adverse health outcomes |
| Nørgaard, A. | 2017 | Psychotropic Polypharmacy in Patients with Dementia: Prevalence and Predictors |
| Onder, G. | 2013 | Polypharmacy and mortality among nursing home residents with advanced cognitive impairment: Results from the shelter study |
| Orsel, K. | 2018 | Psychotropic drugs use and psychotropic polypharmacy among persons with Alzheimer's disease |
| Platen, M. | 2021 | Prevalence of Low-Value Care and Its Associations with Patient-Centered Outcomes in Dementia |
| Platen, M. | 2022 | Associations Between Low-Value Medication in Dementia and Healthcare Costs |
| Sönnerstam, E. | 2016 | Inappropriate Prescription and Renal Function Among Older Patients with Cognitive Impairment |
| Valladales-Restrepo, L. F. | 2019 | Potentially inappropriate prescriptions of anticholinergics drugs in Alzheimer's disease patients |
| Thorpe, J. M. | 2017 | Dual Health Care System Use and High-Risk Prescribing in Patients With Dementia: A National Cohort Study |
| Not reported prevalcence of PIMs or polypharmay in older with dementia (21) |  |  |
| Alzner, Reinhard | 2016 | Polypharmacy, potentially inappropriate medication and cognitive status in Austrian nursing home residents: results from the OSiA study |
| Barton, C. | 2008 | Contraindicated medication use among patients in a memory disorders clinic |
| Bonfiglio, V. | 2019 | Potentially Inappropriate Medications and Polypharmacy: A Study of Older People with Mild Cognitive Impairment and Mild Dementia |
| Chang, Woody | 2018 | Use of Clinical Video Telehealth as a Tool for Optimizing Medications for Rural Older Veterans with Dementia |
| Epstein, N. U. | 2010 | Differences in medication use in the Alzheimer's disease neuroimaging initiative: analysis of baseline characteristics |
| Griffiths, A. W. | 2019 | Pro re nata prescribing and administration for neuropsychiatric symptoms and pain in long-term care residents with dementia and memory problems: a cross-sectional study |
| Kristensson, J. H. | 2021 | Medications causing potential cognitive impairment are common in nursing home dementia units - A cross-sectional study |
| Maclagan, L. C. | 2017 | Frailty and Potentially Inappropriate Medication Use at Nursing Home Transition |
| Nygaard, H. A. | 1999 | Drug use in homes for the aged. A comparison between mentally intact and mentally impaired residents |
| Nygaard, H. A. | 2003 | Nursing-home residents and their drug use: a comparison between mentally intact and mentally impaired residents - The Bergen District Nursing Home (BEDNURS) study |
| Rongen, Sara | 2016 | Potentially inappropriate prescribing in older patients admitted to psychiatric hospital |
| Scheel, J. | 2022 | Predictors of falls and hospital admissions in people with cognitive impairment in day-care: role of multimorbidity, polypharmacy, and potentially inappropriate medication |
| Silay, K. | 2017 | Charlson Comorbidity Index, inappropriate medication use and cognitive impairment : Bermuda Triangle |
| Sonnerstam, E. | 2022 | Potentially Inappropriate Medications Pre- and Post-Diagnosis of Major Neurocognitive Disorders Among Older People in Sweden: A Register-Based, 6-Year Longitudinal Study |
| Sönnerstam, E. | 2023 | Potentially inappropriate medications among elderly people with neurocognitive disorders - A nationwide register-based study using 3 different explicit criteria |
| von Renteln-Kruse, Wolfgang | 2015 | Geriatric Patients With Cognitive Impairment |
| Blass, D. M. | 2008 | Medication use in nursing home residents with advanced dementia |
| Pearson, Scott M. | 2021 | Implementation of Pharmacist Reviews to Screen for Potentially Inappropriate Medications in Patients With Cognitive Impairment |
| Disalvo, D. | 2018 | Potentially Inappropriate Prescribing in Australian Nursing Home Residents with Advanced Dementia: A Substudy of the IDEAL Study |
| Sönnerstam，E | 2017 | An evaluation of the prevalence of potentially inappropriate medications in older people with cognitive impairment living in Northern Sweden using the EU(7)-PIM list |
| Silva-Almodóvar，A | 2020 | Impact of Automated Targeted  Medication Review Electronic Alerts  to Reduce Potentially Inappropriate  Medication Prescribing Among Medicare  Enrolled Patients With Dementia |
| Non-English articles (2) |  |  |
| Manabe, T. | 2019 | Effects of drug treatment on the surviral-time in patients with dementia |
| Martinez Arrechea, S. | 2021 | Prevalence of prescription of anticholinergic/sedative burden drugs among older people with dementia living in nursing homes |
| Repeat (3) |  |  |
| Cross, A. J. | 2017 | Potentially Inappropriate Medication, Anticholinergic Burden, and Mortality in People Attending Memory Clinics |
| Wucherer, D. | 2017 | Drug-related problems in community-dwelling primary care patients screened positive for dementia |
| Hyttinen, V. | 2017 | Incident Use of a Potentially Inappropriate Medication and Hip Fracture in Community-Dwelling Older Persons With Alzheimer's Disease |

| **Section/topic** | **#** | **Checklist item** | **Reported on page #** |
| --- | --- | --- | --- |
| **TITLE** | | |  |
| Title | 1 | Identify the report as a systematic review, meta-analysis, or both. | 1 |
| **ABSTRACT** | | |  |
| Structured summary | 2 | Provide a structured summary including, as applicable: background; objectives; data sources; study eligibility criteria, participants, and interventions; study appraisal and synthesis methods; results; limitations; conclusions and implications of key findings; systematic review registration number. | 1 |
| **INTRODUCTION** | | |  |
| Rationale | 3 | Describe the rationale for the review in the context of what is already known. | 1-3 |
| Objectives | 4 | Provide an explicit statement of questions being addressed with reference to participants, interventions, comparisons, outcomes, and study design (PICOS). | 3 |
| **METHODS** | | |  |
| Protocol and registration | 5 | Indicate if a review protocol exists, if and where it can be accessed (e.g., Web address), and, if available, provide registration information including registration number. | 3 |
| Eligibility criteria | 6 | Specify study characteristics (e.g., PICOS, length of follow-up) and report characteristics (e.g., years considered, language, publication status) used as criteria for eligibility, giving rationale. | 3 |
| Information sources | 7 | Describe all information sources (e.g., databases with dates of coverage, contact with study authors to identify additional studies) in the search and date last searched. | 3 |
| Search | 8 | Present full electronic search strategy for at least one database, including any limits used, such that it could be repeated. | 3 |
| Study selection | 9 | State the process for selecting studies (i.e., screening, eligibility, included in systematic review, and, if applicable, included in the meta-analysis). | 3-4 |
| Data collection process | 10 | Describe method of data extraction from reports (e.g., piloted forms, independently, in duplicate) and any processes for obtaining and confirming data from investigators. | 3 |
| Data items | 11 | List and define all variables for which data were sought (e.g., PICOS, funding sources) and any assumptions and simplifications made. | 3 |
| Risk of bias in individual studies | 12 | Describe methods used for assessing risk of bias of individual studies (including specification of whether this was done at the study or outcome level), and how this information is to be used in any data synthesis. | 4 |
| Summary measures | 13 | State the principal summary measures (e.g., risk ratio, difference in means). | 4 |
| Synthesis of results | 14 | Describe the methods of handling data and combining results of studies, if done, including measures of consistency (e.g., I^2^) for each meta-analysis. | 4 |

Page 1 of 2

| **Section/topic** | **#** | **Checklist item** | **Reported on page #** |
| --- | --- | --- | --- |
| Risk of bias across studies | 15 | Specify any assessment of risk of bias that may affect the cumulative evidence (e.g., publication bias, selective reporting within studies). | 3-4 |
| Additional analyses | 16 | Describe methods of additional analyses (e.g., sensitivity or subgroup analyses, meta-regression), if done, indicating which were pre-specified. | 4 |
| **RESULTS** | | |  |
| Study selection | 17 | Give numbers of studies screened, assessed for eligibility, and included in the review, with reasons for exclusions at each stage, ideally with a flow diagram. | 4-5, Fig. 1 |
| Study characteristics | 18 | For each study, present characteristics for which data were extracted (e.g., study size, PICOS, follow-up period) and provide the citations. | 5-6, Table 1 |
| Risk of bias within studies | 19 | Present data on risk of bias of each study and, if available, any outcome level assessment (see item 12). | 6, Table S2-S3 |
| Results of individual studies | 20 | For all outcomes considered (benefits or harms), present, for each study: (a) simple summary data for each intervention group (b) effect estimates and confidence intervals, ideally with a forest plot. | Fig 2-3 |
| Synthesis of results | 21 | Present results of each meta-analysis done, including confidence intervals and measures of consistency. | Fig 2-3 |
| Risk of bias across studies | 22 | Present results of any assessment of risk of bias across studies (see Item 15). | 6 |
| Additional analysis | 23 | Give results of additional analyses, if done (e.g., sensitivity or subgroup analyses, meta-regression [see Item 16]). | 6-7, Table 2 |
| **DISCUSSION** | | |  |
| Summary of evidence | 24 | Summarize the main findings including the strength of evidence for each main outcome; consider their relevance to key groups (e.g., healthcare providers, users, and policy makers). | 7-8 |
| Limitations | 25 | Discuss limitations at study and outcome level (e.g., risk of bias), and at review-level (e.g., incomplete retrieval of identified research, reporting bias). | 10 |
| Conclusions | 26 | Provide a general interpretation of the results in the context of other evidence, and implications for future research. | 10 |
| **FUNDING** | | |  |
| Funding | 27 | Describe sources of funding for the systematic review and other support (e.g., supply of data); role of funders for the systematic review. | 10-11 |

*From:*  Moher D, Liberati A, Tetzlaff J, Altman DG, The PRISMA Group (2009). Preferred Reporting Items for Systematic Reviews and Meta-Analyses: The PRISMA Statement. PLoS Med 6(7): e1000097. doi:10.1371/journal.pmed1000097

For more information, visit: **www.prisma-statement.org**.
